# Comparison of two brief mindfulness interventions for anxiety, stress and burnout in mental health professionals: a randomised crossover trial

**DOI:** 10.3389/fpsyg.2023.1160714

**Published:** 2023-05-12

**Authors:** Raquel Ruiz-Íñiguez, Ana Carralero Montero, Francisco A. Burgos-Julián, Justo R. Fabelo Roche, Miguel Á. Santed

**Affiliations:** ^1^Faculty of Psychology, Universidad Nacional de Educación a Distancia (UNED), Madrid, Spain; ^2^Department of Nursing, Faculty of Medicine and Health Sciences, Universidad de Alcalá de Henares (UAH), Madrid, Spain; ^3^Center for Academic Development in Drug Addiction (CEDRO), Universidad de Ciencias Médicas de La Habana (UCM-H), Havana, Cuba

**Keywords:** crossover study, meditation, mindfulness, anxiety, stress, burnout, mental health professional, occupational health

## Abstract

**Background:**

Anxiety, stress and burnout are a growing reality among mental health professionals, impacting negatively on them and their clients. Mindfulness-based interventions (MBIs) have demonstrated effectiveness in mitigating these sufferings. Nevertheless, there is a lack of knowledge on the impact of MBIs in Cuba.

**Objectives:**

To compare the effectiveness of two brief mindfulness-based interventions for reducing anxiety, work stress and burnout.

**Methods:**

A total of 104 mental health professionals from Havana (Cuba) participated in a randomised crossover trial. Group A received first an intervention involving body-centred practices (body scan and Hatha yoga) and a second intervention involving mind-centred practices (focused attention and open monitoring meditation). Group B received the same interventions but in reverse order. Four measures (anxiety, stress, burnout syndrome, and antecedents of burnout) were measured at baseline, posttest1, posttest2, and 6-months follow-up.

**Results:**

After the first intervention, there was a between-group difference for burnout syndrome, but the ES was similar for both groups. After the second intervention (implementing both practises), groups showed the largest effect sizes, and there was a between-group difference for antecedents of burnout. Results were partially maintained at 6-month follow-up.

**Conclusion:**

These results suggest that mind-centred practises can be as effective as body-centred practises for stress, anxiety and burnout reduction. The combination of both types of practises could be the most effective way of teaching mindfulness. About the sequence of implementation, teaching mind-centred practises first and then body-centred practises could be most effective for reducing antecedents of burnout.

**Clinical Trial Registration**: www.clinicaltrials.gov NCT03296254.

## 1. Introduction

Anxiety, work stress and burnout are a growing reality among healthcare professionals who are in direct contact with clients, affecting their health conditions and reducing their quality of life (Çelmeçe and Menekay, 2020). The consequences are negative not only for them, but also for people they serve and the institutions they work for (International Labor Organization, 2016). In addition to common job stressors with other health professionals, mental health professionals experience unique stressors. Client suicide, a particularly demanding relationship with people with a high level of psychic suffering, and difficult interactions with other mental health professionals, as they usually work in multidisciplinary teams, are some of these specific stressors (Rössler, 2012).

The practice of mindfulness is inherited from Buddhist meditative practises. Probably the most widespread definition of mindfulness was given by Kabat-Zinn (1994) referring to paying attention on purpose, in the present moment and non-judgmentally. There are a large number of studies on the efficacy of mindfulness-based interventions (MBIs) for improving stress-related outcomes in clinical and non-clinical populations: Goyal et al. (2014) reported moderate evidence to improve anxiety but low evidence to improve stress and mental health; Khoury et al. (2015) reported large effects on stress, moderate effects on anxiety and small effects on burnout; and Lomas et al. (2018) pointed out that MBIs were associated with decreased stress and anxiety but with equivocal results for burnout. Studies and reviews conducted on the impact of MBIs on healthcare professionals have similar results, reporting improvements in stress and anxiety, but the impact on burnout remains unclear due to conflicting conclusions (Rudaz et al., 2017; Suyi et al., 2017; Janssen et al., 2018; Spinelli et al., 2019; Green and Kinchen, 2021; Kriakous et al., 2021). With regard to follow-up periods, a Cochrane review in health professionals (Van Wyk and Pillay-van Wyk, 2010) concluded that there was insufficient evidence on the effectiveness of MBIs and other interventions for stress management and burnout prevention beyond the intervention period.

Although the practice of mindfulness is relevant for healthcare professionals, the time commitment is an important barrier to adherence (Schroeder et al., 2018). For this reason, brief mindfulness interventions (≤30 min or less per session, ≤100 min per week, and/or ≤8 weeks) are being introduced. There is evidence that brief-MBIs can impact numerous health-related outcomes (Howarth et al., 2019); for healthcare providers, studies have reported positive changes in levels of stress, anxiety, and burnout symptoms, among others (Gilmartin et al., 2017).

Mindfulness-based interventions refer to a set of combined exercises in which the participants are instructed. Most of MBIs are based on Mindfulness-Based Stress Reduction (MBSR; Kabat-Zinn, 1982) and mindfulness-based cognitive therapy (MBCT; Segal et al., 2002), the two main standardized protocols. These protocols include three main formal practices: body scan, Hatha yoga and meditation. Body scan practice consists of directing the attention to each body part, one at a time, feeling each part. Hatha yoga integrated a series of smooth and slow movements and stretching, paying attention to every change and pose. Body scan and Hatha yoga are incorporated in MBIs along with classical meditation exercises based on *Vipassana* meditation, confirming MBIs to be a combination of mental training and meditation. Classical meditation exercises included focused attention meditation, which involves sustained attention on a selected object, and open monitoring meditation, which involves observing consciousness itself from moment to moment without reactivity. These three practises are related to each other, as mind and body are powerfully connected. Nevertheless, it has been pointed out that body scan and Hatha yoga are particularly interconnected; both are intimately tied by establishing a way to observe body sensations in which the body is in complete contact with the physical world (Dreeben et al., 2013). Considering the above and in order to avoid the confusion of labels, body scan and Hatha yoga will be classified as body-centred practises and classical meditation as mind-centred practises in this study. Regarding the order in which to teach the different practises, body scan is the first formal practice performed in MBSR, and is the initial encounter with mindfulness (Dreeben et al., 2013). Cebolla and Campos (2016) have also recommended instruction in body scan and mindful movements before advancing to meditation when teaching mindfulness.

There are several conceptual and methodological problems that need to be addressed in MBI research. The multidimensionality of MBI studies (using a range of mindfulness techniques and practises combined) has been pointed out as a particular problem in mindfulness research, highlighting the need to study the types of components and practises (Davidson and Kaszniak, 2015; Demarzo et al., 2017). The studies preclude understanding the effects of the specific practises (Britton et al., 2018), so they are limited in the ability to know whether the change is due to a specific component of mindfulness or to multi-component synergy (Chiesa and Malinowski, 2011). Thus, it is of interest to examine whether some practises are more strongly related to some treatment results than others; furthermore, the duration of the MBIs and the most effective sequence of the practises should be noted (Demarzo et al., 2017; Parsons et al., 2017). Hence, elucidating the active components of MBIs is an important step in order to validate them and to refine models of the mechanisms of action of mindfulness (Stein and Witkiewitz, 2020).

The evidence points to yoga and mindful movements as being effective interventions for reducing stress and anxiety in non-clinical population (Robert-McComb et al., 2015; Fishbein and Saper, 2019). Call et al. (2014) studied the reduction of stress and anxiety through yoga and body scan in female undergraduate students, concluding that both practises were effective at reducing anxiety and stress symptoms. A qualitative study (Colgan et al., 2017) conducted on war veterans found a higher percentage of people who reported feeling decreased reactivity to stress using body scan (72%) compared to mindfulness of breath meditation (57%), which was the main focused attention meditation practice. Hilcove et al. (2021) studied the effects of a mindfulness-based yoga practice on stress, burnout and well-being among nurses and healthcare professionals, with significant improvements in all three conditions.

It should be noted that dismantling studies are best suited for determining whether there is a particular component critical to treatment benefits (Baskin et al., 2003). In this regard, there are a few mindfulness studies. One systematic review focused on dismantling MBIs for a clinical population (Stein and Witkiewitz, 2020) and included studies on testing the components of MBIs. Most of the included studies focused on dismantling by comparing the standardized MBI with a version of MBI without the active ingredient (practice) or without some training skills (e.g., acceptance); only one study (Britton et al., 2018) actually focused on dismantling classical mindfulness meditation exercises (i.e., focused attention vs. open monitoring). Hunt et al. (2018) dismantled yoga and mindfulness training (based on informal meditation, body scan and sitting meditation) in an MBI for college students. In a meta-analysis by Eberth and Sedlmeier (2012), they conducted a sub-analysis of the differences between MBSR and just mindfulness meditation exercises. When dismantling studies, non-specific aspects of the interventions (e.g., the link with the facilitator, the user’s expectations or the clinical environment) must be considered to ensure equivalence across groups (Proulx, 2003; Papa and Follette, 2015).

No study found to date has investigated structurally equivalent MBIs, differing only in the formal mindfulness exercises, by comparing body-centred practises and mind-centred practices. In this study, we therefore focus on that issue. Furthermore, MBIs require an active role by the participants, with home practice being a fundamental component of the intervention (Parsons et al., 2017). Thus, compliance and previous experience are key issues to be addressed (Parsons et al., 2017; Kriakous et al., 2021).

The objective of the study was to compare the effectiveness of two brief MBIs, one involving body-centred practises (body scan and Hatha yoga) and another involving mind-centred practises (focused attention and open monitoring meditation) in reducing levels of anxiety, work stress and burnout in Cuban mental health professionals. Mindfulness is still little known by mental health professionals in Cuba, making it an ideal context for research. It was hypothesised that: (1) mind-centred practice alone would be less effective than body-centred practice alone; (2) adding body-centred practice to mind-centred practice would be less effective than adding mind-centred practice to body-centred practice (immediately after); (3) training in the two kind of practises, combined one after the other, would be significant for all outcome variables in both groups, with higher effect sizes (ES) for stress and anxiety than for burnout; (4) the effectiveness will remain at 6 months of follow-up for all outcome variables in both groups.

## 2. Materials and methods

### 2.1. Participants

Participants were recruited from November to December 2017. A non-research assistant informed to potential participants of a mindfulness training through mailing lists of key people (professionals of repute, coordinators, and heads of services) and through a call on the Infomed website (portal of the Cuban Health Network) for professionals working in the mental health services of Havana province (Cuba). The inclusion criteria were: (1) to be a mental health professional in active employment; (2) delivering direct patient care; and (3) committed to meet the requirements for attendance and dedication to the course. The exclusion criteria were to suffer from a general medical condition or a mental disorder that discourages participation in the study.

The sample size calculation was based on the four primary outcomes as a composite outcome. *A priori* power analysis based to achieve a large effect size (Cohen’s *f* = 0.40), following the recommendations of Brysbaert and Stevens (2018), revealed that 70 participants were needed for testing the null-hypothesis of equality (*α* = 0.05) with a power of 80% in a two-group, four-timepoint design (ANOVA 2 × 4).

**Figure 1:**
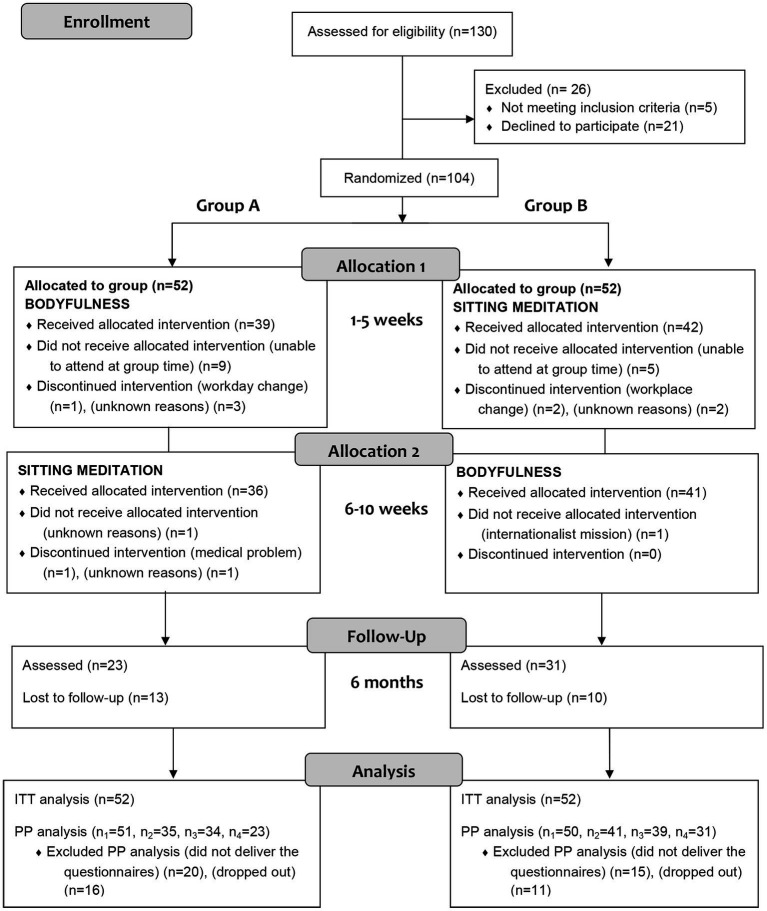
CONSORT flow diagram.

The initial sample consisted of 130 participants; out of this, 104 were randomised in the two experimental groups. Figure 1 shows the flowchart of the study.

### 2.2. Procedure

Participants were assigned into two experimental groups (A and B) in a crossover design. The randomization sequence was created using Excel 2010 (Microsoft, Redmond, WA, United States), with a 1:1 allocation by a non-research assistant. After being accepted as part of the research, the participants were assigned to interventions by the assistant and then informed of the day and time of attendance at the course. The assistant was also the contact person for reporting any problems before the beginning of the interventions. Investigators were blinded to the allocation during the process and the instructor (first author) did not have access to the participant lists of each group until the first day of sessions.

The interventions ran from January to March 2018. Group A first received an intervention of five meditation sessions focused on body-centred practises while Group B first received an intervention of five meditation sessions focused on mind-centred practices. From the sixth session, Group A second received the mind-centred practises intervention and Group B received the body-centred practises intervention. Therefore, each group attended a total of 10 sessions. There was also a final extra non-meditative session, as a farewell meeting, following the customs of the country. Assessments were administered at four time points: at the beginning of the initial session (i.e., prepost, week 1), at the beginning of the sixth session (i.e., posttest1, week 6), at the beginning of the farewell session (i.e., posttest2, week 11), and 6 months after the 10th session (i.e., follow-up, week 37–39).

### 2.3. Interventions

The intervention instructor (first author) for both interventions was a mental health nurse expert in mindfulness, certified by the *Spanish Association of Mindfulness and Compassion* (AEMIND), and with more than 7 years of mindfulness training. Another professional (last author), a clinical psychologist with more than 13 years of experience as an instructor, supported the implementation. Together, they elaborated the content of the programs of both interventions, guided by the works of Kabat-Zinn (2005) and Segal et al. (2002). Since mindfulness training is currently beginning in Cuba, an on-the-spot qualified instructor to carry out the intervention instead of the researcher was not found. Each intervention comprises 5 weekly face to face group sessions of 2.5 h. Thus, each group received a 10-week global training. They were structurally equivalent and differed only in the active ingredient of their content (i.e., formal mindfulness exercises). Body scan and Hatha yoga were the formal practises included in the body-centred intervention. Focused attention and open monitoring meditation were the formal practises included in the mind-centred intervention.

To ensure the equivalence of the interventions, considerations made by Davidson and Kaszniak (2015) were assumed. Thus, both interventions had the same structure, frequency, duration and theoretical content; classes were similar in size and held in the same location; the same amount of practice was required, both in sessions and in daily home practice; and the instructor of both was the same. All participants were urged to practice at home 6 times/week, at least for 20 min/day, this being the duration of each practice in the audio guides provided and conducted by the instructor. All participants were also instructed in informal meditation, since it is widely incorporated in MBIs. Informal meditation was presented as a complementary practice to do at home and the exercises were the same for both interventions. See the Supplementary material for a detailed description of the structuring of the sessions (Supplementary Table S1), and the formal practice and homework for both interventions (Supplementary Tables S2, S3, respectively).

### 2.4. Measures

For the perception of work stressors, the Spanish Symptomatic Stress Scale (SSS) of Aro (1981) was used, in its version of the National Institute of Cuban Workers of 1983 (Carballo Herrera et al., 2014; Hernández Almirall and Marroquín, 2016). It contains 18 items, which respond to symptoms that are usually associated with stress states, of a psychosomatic, emotional or cognitive nature. The responses are articulated on a Likert-type scale from 0 to 3, with scores between 0 and 54. It establishes two levels for the participant: unaffected by stress (≤10 points) and affected by stress (>10 points). The internal consistency reliability measure of this and the following instruments was performed using McDonald’s omega coefficient, a statistic recommended as a substitute for Cronbach’s alpha when the data is ordinal (Revelle and Condo, 2018). In this study, the McDonald’s omega was 0.81.

To determine the state of anxiety, the Spanish state anxiety inventory (STAI-S) was used (Spielberger and Díaz-Guerrero, 1975). It has 20 items and has been validated in the Cuban population (González Llaneza, 2007b). It is a self-assessment questionnaire designed to evaluate anxiety as a transient emotional condition. The responses are articulated on a Likert-type scale from 1 to 4, with scores between 20 and 80. A score of 45 was established as a cut-off point for high anxiety, and a score of 34 for low anxiety. In this study, the McDonald’s omega was 0.93.

The Brief Burnout Questionnaire (BBQ) was used to measure burnout (Jiménez et al., 1997), which is an adaptation of Maslach Burnout Inventory and has a content validation for Cuban population(González Llaneza, 2007a). The BBQ has 21 items and measures the burnout syndrome and its antecedents (i.e., the perception of certain external aspects that lead the person to develop the syndrome, considering: tedium, characteristics of the task, and organisation environment). It is answered on a Likert-type scale from 1 to 5. Burnout syndrome is categorised as low (9–19 points), medium (>19–25 points), and high (>25 points). In this study, the McDonald’s omega was 0.77 for syndrome and 0.82 for antecedents.

The degree of compliance was determined by the minutes of performed practises (at face-to-face sessions and at home). For home practice, participants were provided with a weekly practice log, in which they must specify the time spent (in minutes) for both formal and informal practice per day. Crane et al. (2014) pointed out that the use of the imputation of missing data in the analysis of home practice is not an appropriate option, since it would overestimate the figures. Therefore, when the practice log was not returned or left blank, it was counted as *Zero minutes of practice* in that week, following the considerations in previous studies (Crane et al., 2014; Quach et al., 2017).

Data about prior expectations and previous experience was collected to ensure the equivalence of the samples. Expectations were assessed both personally and professionally. A single item for each one was used: *Do you hope that the practice of mindfulness will help you improve your level of personal/work well-being?* with five possible responses on a Likert-type scale (being 1 *nothing* and 5 *a lot*). Experience in related techniques was investigated with the open question: *What similar techniques have you practised?* Participants mentioned transcendental meditation, Taichi, Chi Kung, and yoga. In addition, certain sociodemographic data included in the BBQ were collected: gender, age, relationship (with/without a partner), profession, years in profession and workplace.

### 2.5. Statistical analysis

A multivariate linear model was estimated. Firstly, the main effects of the group, time and group × time interaction factors were analysed by multivariate analysis of variance (MANOVA). This model allows several dependent variables to be estimated jointly, calculating a theoretical value based on the values of these variables weighted by the weight of each one of them (Hair et al., 2014). The analysis included *stress*, *anxiety*, *burnout syndrome* and *antecedents of burnout* as the four dependent variables and group factor (A and B) and time (pretest, posttest1, posttest2 and follow-up) as independent variables. Secondly, if statistically significant differences in the main factors or in the interaction were found from the MANOVA, the corresponding univariate analyses (ANOVA 2 × 4) were estimated. Thirdly, if statistically significant results were found in the ANOVA, the corresponding *post hoc* tests were estimated. We calculated the *post hoc* test for the significant factors using Holm’s criterion for the value of *p* correction (Aickin and Gensler, 1996). To test the validity of the design, an analysis of the carry-over effects was included. The sum of the measured values obtained at the end of the components of each intervention inside of each group was used, comparing these results using the Student’s *t*-test, for independent samples (Wellek and Blettner, 2012).

The ES associated with the analysis of variance were reported using *ω*^2^ and for *post hoc* tests we used Cohen’s *d*. Following the current recommendations (Solla et al., 2018), the interpretation of the significance of the results was performed using both the significance level (value of *p*) and the ES and its confidence interval, whose absence of the zero value determines the presence of statistical significance. Data analysis was performed based on the basis of an intention-to-treat (ITT) analysis, using the imputation procedure based on the expected maximisation method (Dempster et al., 1977). Likewise, the results of the multivariate linear model estimated by per-protocol (PP) analysis were reported. The analyses were carried out using R software (RRID: SCR_001905).

## 3. Results

### 3.1. Baseline characteristics and compliance

The final sample was made up of 90% women and an average of 41 years (*SD* = 11.91; range = 20–66). Descriptive statistics and between-group comparisons appear in Table 1. Most of the participants (63.1%) did not have previous experience in techniques related to mindfulness. Expectations about the improvement that training would bring to them were high in both groups, both related to their personal well-being (*M*_A_ = 4.33, *SD*_A_ = 0.96; *M*_B_ = 4.58 *SD*_B_ = 0.64) and related to their work welfare (*M*_A_ = 4.17, *SD*_A_ = 0.98; *M*_B_ = 4.44, *SD*_B_ = 0.67). No statistically significant intergroup differences were found either in previous experience or in the level of expectation (*p-*values >0.01). At baseline, there were no statistically significant differences in the sociodemographic and employment data (see Table 1) nor in the outcome variables (see Table 2).

**Table 1:** Descriptive statistics and between group comparisons of sociodemographic and labor data.

	*N^a^*		Total	*n*^†^	Group A	*n*^†^	Group B	*t*/*χ*^2^ value	*p*
Age, *M (SD)*	103		40.9 (11.9)	51	39.4 (11.4)	52	42.5 (12.3)	-1.31	0.19
Women, *n* (%)	103		93 (90.3)	51	43 (84.3)	52	50 (96.1)	4.12	0.05
With couple, *n* (%)	103		80 (77.7)	51	43 (84.3)	52	37 (71.1)	2.58	0.28
Profession, *n* (%)	102	Psychology	56 (54.9)	51	31 (60.8)	51	25 (49)	1.72	0.42
		Psychiatry	35 (34.3)		16 (31.4)		19 (37.2)		
		Nursing	13 (10.8)		4 (7.8)		7 (13.7)		
Years in profession, *M (SD)*	101		10.9 (9.7)	50	9.9 (8.9)	51	12.01 (10.5)	-1.11	0.27
Workplace, *n* (%)	102	Hospital	51 (50)	51	24 (47.06)	51	15 (29.4)	3.36	0.07
		Primary	51 (50)		27 (52.9)		36 (70.6)		

† Values vary due to lost data.

Regarding compliance, the completers comprised 71.2%. More than a half of dropouts (51.85%) occurred after knowing the allocation of the weekday for face-to-face group sessions (see Figure 1). Absent at ≥3 sessions (out of 5) in the same intervention were considered as dropout. The follow-up rate was 70.13% (out of 77 completers). All the completers attended at least 60% of the sessions, with an average attendance of 4 sessions (out of 5) sessions in both groups. No statistically significant differences were found between dropouts and completers for any of the baseline states of the outcome variables (all *p-*values > 0.01). Regarding the face-to-face attendance, the average of minutes of formal practice was 304.7 min (*SD* = 101.54), with a wide range from 45 to 390 min, from a maximum minute of 430 (215 min in total for each intervention). No significant intergroup differences were detected in the minutes of practice in the face-to-face sessions (all *p-*values > 0.01). At the end of the 10 weeks, the mean time devoted to home practice was 261.30 min (*SD =* 201.06) for body-centred intervention and 346.41 min (*SD =* 248.90) for mind-centred intervention, from a maximum of 600 min per intervention. No statistically significant intergroup differences were found in the amount of home practice, both formal and informal practice (all *p* values > 0.01). All participants who attended follow-up were still formally practising; 54.72% of them regularly, i.e., three or more times a week (Group A: 47.83%; Group B: 62.07%), with no significant intergroup differences (*p-*values > 0.01).

### 3.2. Between-group and within-group comparisons

In the MANOVA (Table 3), statistically significant differences were reported for the four dependent variables (bundled together into a composite variable). The differences were found between groups (A and B) for both ITT and PP analysis (*p* < 0.01) and over time (at some point) for both ITT and PP analysis (*p* < 0.01). No statistically significant interaction effects were found (*p* > 0.01). Therefore, subsequent analyses did not include the interaction term.

**Table 3:** MANOVA results.

Factor	ITT analysis			PP analysis		
	F^†^	p	𝝎^2^	F^†^	*p*	𝝎^2^
Group	5.13	<0.01*	0.04	4.74	<0.01*	0.03
Time	5.93	<0.01*	0.12	3.14	<0.01*	0.06
Interaction	0.04	0.59	0.00	1.34	0.15	0.01

† Pillai’s trace.

* *p* < 0.05.

𝝎^2^ ~ 0.01 E.S. small; 𝝎^2^ ~ 0.06 E.S. medium; 𝝎^2^ ~ 0.14 E.S. large.

ITT, intention-to-treat; PP, per-protocol.

Next, we carried out ANOVA analyses to determine in which dependent variables the differences were. Missing data analysis showed an average of 28.8% missing data for the set of dependent variables and timepoints. ITT analyses are considered to be superior to PP analyses because PP analyses only achieve results as reliable as ITT analysis when the percentage of missing data is not greater than 5% (Madley-Dowd et al., 2019); besides, no difference between ITT and PP analyses were found in the results for the MANOVA. Thus, only the ITT analysis results are presented. Statistically significant differences between groups were found (Table 4), with a small ES, for *burnout syndrome* (*ω*^2^ = 0.03) and for *antecedents of burnout* (*ω*^2^ = 0.05), and significant within-group differences were found for all outcome variables, with ESs from *ω*^2^ = 0.26 (*antecedents of burnout*) to *ω*^2^ = 0.54 (*stress*).

**Table 4:** Univariant ANOVA results, main effects, group and time (ITT analysis).

Measure	Group				Time			
	*F*	*p*	𝝎^2^	IC90%	*F*^†^	*p*	𝝎^2^	IC90%
SSS	0.01	0.95	0	[0, 1.00]	42.22	<0.01^*^	0.54	[0.47, 0.60]
STAI-S	0.14	0.71	0	[0, 1.00]	26.91	<0.01^*^	0.43	[0.35, 0.49]
BBQ. Syndrome	4.79	0.03^*^	0.03	[0, 0.11]	36.96	<0.01^*^	0.51	[0.44, 0.57]
BBQ. Antecedents	7.01	0.01^*^	0.05	[0.01, 0.14]	13.33	<0.01^*^	0.26	[0.19, 0.33]
BBQ. Consequences	0.24	0.62	0	[0, 1.00]	17.07	<0.01^*^	0.32	[0.24, 0.38]

† Greenhouse – Geisser.

* *p* < 0.05.

𝝎^2^ ~ 0.01 E.S. small; 𝝎^2^ ~ 0.06 E.S. medium; 𝝎^2^ ~ 0.14. E.S. large.

SSS, symptomatic stress scale; BBQ, brief burnout questionnaire; STAI-S, anxiety-state inventory.

The between-group *post hoc* analysis (Table 2) found statistically significant differences for *burnout syndrome* at posttest1 (after completed the first intervention) and posttest2 (after completed the two interventions), with a medium ES (*d* = 0.43 and *d* = 0.41, respectively); and for *antecedents of burnout* at posttest2 and follow-up, with a medium ES (*d* = 0.56, *d* = 0.62, respectively).

**Table 2:** Means, standard deviations and between group differences at each time point.

	Total (*N* = 104)	Group A (*n* = 52)	Group B (*n* = 52)	*t*	*p*	*d*	IC 95%
	*M (SD)*	*M (SD)*	*M (SD)*				
SSS							
Pretest	6.75 (4.36)	6.92 (4.70)	6.58 (4.04)	0.39	0.69	0.08	[–0.31,0.47]
Posttest1	5.64 (3.84)	5.89 (3.71)	5.39 (3.99)	0.66	0.51	0.13	[–0.26,0.52]
Posttest 2	3.60 (2.89)	3.61 (2.94)	3.59 (2.86)	0.045	0.96	0.01	[–0.38,0.40]
Follow-up	3.96 (2.70)	3.61 (2.29)	4.31 (3.04)	–1.33	0.18	–0.26	[–0.65,0.13]
STAI-S							
Pretest	31.89 (7.07)	30.89 (5.61)	32.89 (8.22)	–1.45	0.15	–0.28	[–0.67,0.11]
Posttest1	30.04 (5.65)	29.98 (6.37)	30.10 (4.89)	–0.11	0.91	–0.02	[–0.41,0.37]
Posttest 2	26.00 (4.34)	25.69 (4.60)	26.31 (4.07)	–0.72	0.47	–0.14	[–0.53,0.25]
Follow-up	30.74 (7.15)	31.49 (7.68)	30.00 (6.56)	1.63	0.29	0.21	[–0.18,0.60]
BBQ. Syndrome							
Pretest	19.76 (4.52)	20.58 (4.24)	18.97 (4.71)	1.89	0.06	0.36	[–0.03,0.75]
Posttest1	18.56 (3.56)	19.31 (3.42)	17.81 (3.56)	2.19	0.03^*^	0.43	[0.04,0.82]
Posttest 2	17.13 (3.34)	17.80 (3.49)	16.45 (3.07)	2.09	0.04^*^	0.41	[0.02,0.80]
Follow-up	17.00 (2.81)	17.34 (2.65)	16.66 (2.96)	1.23	0.22	0.24	[–0.15,0.63]
BBQ. Antecedents							
Pretest	17.22 (4.34)	18.01 (4.34)	16.43 (4.23)	1.87	0.06	0.37	[–0.02,0.76]
Posttest1	16.70 (4.08)	17.35 (4.16)	16.05 (3.94)	1.64	0.10	0.32	[–0.07,0.71]
Posttest 2	15.42 (3.62)	16.40 (3.45)	14.45 (3.54)	2.85	0.01^*^	0.56	[0.16,0.95]
Follow-up	16.14 (3.67)	17.23 (3.91)	15.06 (3.09)	3.45	<0.01^*^	0.62	[0.22,1.01]

* *p* < 0.05.

*d* Cohen: *d* ~ 0.20 E.S. small; *d* ~ 0.50 E.S. medium; *d* ~ 0.80 E.S. large.

SSS, symptomatic stress scale; BBQ, brief burnout questionnaire; STAI-S, anxiety-state inventory.

Therefore, there was a difference in effectiveness between body- and mind-practises for *burnout syndrome* and there was a difference in effectiveness depending on the order of implantation of the two kind of practises for *burnout syndrome* and *antecedents of burnout*. Finally, there was difference in effectiveness over time for *antecedents of burnout.*

In the within-group *post hoc* analysis (Table 5), after the first intervention was completed (pretest–posttest1), only Group B (trained in mind-centred practises) produced statistically significant decreases for *anxiety,* with medium ES (*d* = 0.43) and the reduction of *burnout syndrome* was significant in both groups (ES: *d*_A_ = 0.33; *d*_B_ = 0.31). When the two interventions were completed (pretest–posttest2), significant differences were found in both groups for all outcomes for *stress* (*d_A_* = 0.88, *d_B_* = 0.79), *anxiety* (*d_A_* = 0.95, *d_B_* = 0.79), *burnout syndrome* (*d_A_* = 0.72; *d_B_* = 0.70) and *antecedents of burnout* (*d_A_* = 0.49; *d_B_* = 0.73). At 6 months (pretest–follow-up), significant reductions were found for *stress*, with large ES (*d_A_* = 0.89, *d_B_* = 0.90). Significant reductions were also found for *burnout syndrome,* with large ES in Group A (*d* = 0.86) and medium ES in Group B (*d* = 0.63). Significant reductions were found in Group B for *anxiety* and *antecedents of burnout*, with small ES (*d* = 0.31 and *d* = 0.43, respectively). Therefore, both interventions in isolation improved *burnout syndrome* but *anxiety* was improved only by mind-centred practises. The largest impact (medium–large ES) was reached when the two groups had performed both interventions. This improvement was maintained over time for *stress* and *burnout syndrome,* decreasing or becoming non-significant for *anxiety* and *antecedents of burnout.*

**Table 5:** Within-group differences at each time point.

	Pretest-postest1				Pretest-postest2	Pretest-follow up			
	*t*	*p*^†^	*d*	IC 95%	*t*	*p*^†^	*d*	IC 95%	*t*	*p*^†^	*d*	IC 95%
SSS												
Group A	1.75	0.17	0.24	[–0.03, 0.52]	6.32	<0.01^*^	0.88	[0.55, 1.19]	6.39	<0.01^*^	0.89	[0.56, 1.20]
Group B	1.96	0.11	0.27	[–0.01, 0.55]	5.72	<0.01^*^	0.79	[0.48, 1.10]	6.51	<0.01^*^	0.90	[0.58, 1.22]
STAI-S												
Group A	0.96	0.69	0.13	[–0.14, 0.41]	6.85	<0.01^*^	0.95	[0.62, 1.27]	-0.49	0.69	0.07	[-0.34, 0.20]
Group B	3.09	0.01^*^	0.43	[0.14, 0.71]	5.69	<0.01^*^	0.79	[0.47, 1.10]	2.24	0.06	0.31	[0.03, 0.59] ^*^
BBQ. Syndrome												
Group A	2.73	0.01^*^	0.33	[0.10, 0.66]	5.20	<0.01^*^	0.72	[0.41, 1.02]	6.22	<0.01^*^	0.86	[0.54, 1.18]
Group B	2.24	0.06	0.31	[0.03, 0.59]^*^	5.08	<0.01^*^	0.70	[0.40, 1.01]	4.55	<0.01^*^	0.63	[0.33, 0.93]
BBQ. Antecedents												
Group A	1.30	0.39	0.18	[-0.09, 0.45]	3.51	0.01^*^	0.49	[0.20, 0.77]	1.59	0.35	0.22	[-0.06, 0.49]
Group B	0.86	0.39	0.12	[-0.15, 0.39]	5.25	<0.01^*^	0.73	[0.42, 1.03]	3.07	0.01^*^	0.43	[0.14, 0.71]

† *p* corrected value (Holm).

* *p* < 0.05. It is also marked with * the cases *p* ≥ 0.05, *d* > 0.20 and 0 ∉ 95% IC.

*d* Cohen: *d* ~ 0.20 E.S. small; *d* ~ 0.50 E.S. medium; *d* ~ 0.80 E.S. large.

SSS, stress symptomatic scale; BBQ, brief burnout questionnaire; STAI-S, anxiety-state inventory.

The carry-over analysis (Table 6) showed the presence of carry-over effects for *burnout syndrome* and *antecedents*. Thus, an analysis of covariance (ANCOVA) was performed with the carry-over effect analysed as a covariate to control for it. The ANCOVA showed a tendency toward significance for *antecedents of burnout* at posttest2 [*F* (1,101) = 3.22, *p* = 0.07; *ω*^2^ = 0.02, I.C. 95% (0.00, 0.09)], and statistically significant effects for *antecedents of burnout* at follow-up [*F* (1,101) = 4.17, *p* = 0.04; *ω*^2^ = 0.03, I.C. 95% (0.00, 0.10)].

**Table 6:** Estimation of carry-over effects.

Variable	*t*	*p*	*d*	95 % CI^†^
SSS	0.41	0.68	0.08	[–0.30, 0.46]
STAI-S	–0.42	0.67	0.08	[–0.47, 0.30]
BBQ. Syndrome	2.30	0.02^*^	0.46	[0.06, 0.84]
BBQ. Antecedents	2.34	0.02^*^	0.46	[0.07, 0.85]

† Confidence interval for E.S.

* *p* < 0.05.

*d* Cohen: *d*~.20 E.S. small; *d*~.50 E.S. medium; *d*~.80 E.S. large.

SSS, stress symptomatic scale; BBQ, brief burnout questionnaire; STAI-S, anxiety-state inventory.

## 4. Discussion

Firstly, it cannot be concluded that there was a difference in effectiveness between mind- and body-centred practises for stress, anxiety or burnout. Although there was a between-group difference for burnout syndrome at posttest1, the ES was similar for both groups at this timepoint (ES: dA = 0.33; dB = 0.31) and although only mind-centred practises produced significant decreases for anxiety, with medium ES (*d* = 0.43), no between-group differences were found at this timepoint. These results differ from the first hypothesis and show that mind-centred practises could be as effective as body-centred practises, but more studies are necessary to clarify this question. To the best of our knowledge, there are no previous quantitative studies that have compared both interventions with each other. Our results contrast with a qualitative study in which participants felt less stressed practising body scan compared to meditation, although in that study the sample consisted of veterans (Colgan et al., 2017).

Secondly, it can be concluded that the sequence of implementation of the practises produced a change in effectiveness for antecedents of burnout (i.e., tedium, characteristics of the task, and organisation environment). There was a between-group difference found at posttest2, being more effectiveness to start with mind-centred practises followed by body-centred practises than in reverse order (*d_B_* = 0.73; *d_A_* = 0.49). Although there was a between-group difference for burnout syndrome at this timepoint, the ES was similar for both groups (ES: *d*_A_ = 0.72; *d*_B_ = 0.70). We have found no studies that have compared the order of implementation of these practises to contrast these results. Nevertheless, our results reject the habitual starting order, with body scan being the first formal technique to be taught (4,12,36) and contrast with the recommended instruction in body scan and mindful movements before advancing to meditation (36) when teaching mindfulness.

Thirdly, our study indicated that the use of both body- and mind-centred practises combined (posttest2) would be effective for reducing stress, anxiety and burnout, with a greater effect on the first two variables (large ES) compared to burnout (medium–large ES). These results would support the third hypothesis of this study. Our results contrast with recent findings of MBIs that showed that health professionals had the most powerful results, among others, in terms of stress and anxiety, but not in burnout (Janssen et al., 2018; Kriakous et al., 2021). Conversely, our results are in line with a review that found significant results in burnout (Rudaz et al., 2017) and with a review in health professionals, with smaller ES for burnout than for anxiety and stress (Spinelli et al., 2019).

Our results at posttest2 were noticeably better than at posttest1. Mind- and body-centred practises in isolation were not effective, and if they were, it was with a small ES; however, mind- and body-centred practises combined were effective, with a medium or large ES. Assuming that brief MBIs are as effective as standard MBIs (Demarzo et al., 2017; Kriakous et al., 2021), so that 5-week and 10-week interventions show similar effectiveness, our study would provide an important finding: that the use of both body-and mind-centred practises combined would be more effective than using them separately for the outcomes studied. Therefore, the combined exercises used in MBIs would be a crucial aspect of their effectiveness. Our results are in line with the results of a meta-analysis that reported MBSR to be more powerful than classical mindfulness meditation in psychological well-being (Eberth and Sedlmeier, 2012). There are some studies that found significant improvement of stress, anxiety and burnout when practising yoga and/or body scan without mindfulness meditation (Call et al., 2014; Robert-McComb et al., 2015; Fishbein and Saper, 2019; Hilcove et al., 2021) but they did not compare effectiveness. Our results would confirm that the positive impact found in studies carried out on MBIs in health professionals (Suyi et al., 2017; Green and Kinchen, 2021) is related to the use of the practises combined. It has already been pointed out that combined mindfulness training has a slight advantage in some psychological results (Hunt et al., 2018). Mindfulness mechanisms of action would work synergistically, establishing a greater self-regulation process (Hölzel et al., 2011).

Finally, at the 6-month follow-up, the effectiveness remained for stress (large ES) and burnout syndrome (medium-large ES) but decreased for the two other outcomes (small ES). For antecedents of burnout, the effects were only maintained in one group, with statistically significant between-group differences at this timepoint. Nevertheless, the significant carryover effect for antecedents of burnout at this timepoint compromises the assumption of a between-group difference. Our results partially support the last hypothesis of this study and are in line with meta-analytical evidence that has reported decreases in the ES of MBIs over time while still having effectiveness (Goyal et al., 2014; Khoury et al., 2015). Our results contrast with a review that pointed out there was insufficient evidence of effectiveness of MBIs and other stress management interventions in the follow-up periods (Van Wyk and Pillay-van Wyk, 2010).

### 4.1. Strengths and limitations: future research

To our knowledge, this is the first study to assess the differential impact of two brief MBIs on work-related well-being among mental health professionals and the first randomised clinical trial of MBIs in a Cuban sample. It is of interest to deepen and clarify aspects that comprise the multicomponentiality of MBIs: specifically, whether there are differences in the effectiveness of different exercises and differences in the effectiveness according to the order of implementation. It is also important to provide evidence about whether the combination of different types of exercises in MBIs matters in terms of effectiveness. This allows greater flexibility, adapting interventions according to needs.

This study has some limitations. The main limitation was that an investigator provided the interventions, so they could influence the participants to give the desired results (experimenter or Rosenthal effect). However, both groups were experimental and only the researcher doing the interventions knew which interventions the participant was receiving until the trial was over (single-blind study). Nevertheless, it is not enough to mitigated this bias, so our results must be interpreted with caution. The sample was representative for estimating the effects in mental health professionals who decide to participate in mindfulness training; however, our results are not generalizable to professionals not interested in mindfulness or to a clinical sample. There was the limitation of selecting questionnaires validated in Cuba and it should be noted that many participants were psychologists, who are presumably familiar with these questionnaires. The levels of dispositional mindfulness were not measured because there are no validated instruments in the Cuban population, so there could be previous differences in dispositional mindfulness that affect the described findings. Although the results at follow-up were positive, it is not possible to know if this was a direct result of study participation, as there was no control group for comparison. All subjects who responded at follow-up declared that they would keep practising, so the results of those who did not continue practising are unknown. Finally, it may not be the additive benefit of the two kind of practices (body-centred and mind-centred practises) that is important but the total duration of the intervention.

In future research, it would be necessary to use diverse and representative samples in order to generalise the results. It would be interesting to compare interventions based on MBCT instead of MBSR to find out if they yield better results in burnout variables, since burnout syndrome reflects depression more than anxiety. It would also be advisable to include a non-meditative control group and an active control group in which all the exercises are combined following the usual model in a brief MBI. It is necessary to use different measurements, calculating perceptions indirectly through patients and/or participants’ supervisors and to incorporate physiological, neuroendocrine-immune and cognitive measures. Long-term research is needed as well, with interventions including updates or booster sessions that could have a more sustained positive effect.

## 5. Conclusion

It can be concluded that there would be no differences in the effectiveness of mind-centred practises compared to body-centred practises for the management of stress, anxiety and burnout and that a combination of both types of practises would be the most effective. Starting mindfulness training with mind-centred practises first is more effective than starting mindfulness training with body-centred practises first, for the reduction of the antecedents of burnout. After 6 months of follow-up, the effectiveness lessened slightly.

## Data availability statement

The raw data supporting the conclusions of this article will be made available by the authors, without undue reservation.

## Ethics statement

The studies involving human participants were reviewed and approved by Ethics Committee of General Calixto García Faculty of the University of Medical Sciences of Havana (page 168, 2018). The patients/participants provided their written informed consent to participate in this study.

## Author contributions

RR-Í designed and executed the study and drafted the paper. ACM collaborated with the writing of the study and editing of the final manuscript. FB-J analysed the data and commented on the manuscript. JFR obtained regulatory approvals, contributed to the study design and commented on the manuscript. MS participated in the design of the study, monitoring it and revision of the manuscript. All authors contributed to the article and approved the submitted version.

## Conflict of interest

The authors declare that the research was conducted in the absence of any commercial or financial relationships that could be construed as a potential conflict of interest.

## Publisher’s note

All claims expressed in this article are solely those of the authors and do not necessarily represent those of their affiliated organizations, or those of the publisher, the editors and the reviewers. Any product that may be evaluated in this article, or claim that may be made by its manufacturer, is not guaranteed or endorsed by the publisher.
