# Supplementary material for: Comparison of two brief mindfulness interventions for anxiety, stress and burnout in mental health professionals: a randomised crossover trial
Source: Front Psychol. 2023 May 12;14:1160714. doi: 10.3389/fpsyg.2023.1160714 (PMC10214838; doi:10.3389/fpsyg.2023.1160714)
Supplement: Supplementary file 1 [file Data_Sheet_1.docx]

Supplementary Material

Comparison of two brief mindfulness interventions for anxiety, stress and burnout in mental health professionals: A randomized crossover trial

# Supplementary Tables

| **Table S1.** Session planning | | | |
| --- | --- | --- | --- |
| Section | Time | Content | Further explanation |
| Welcome | 15 min | Review of home practice | Letting the participants to explain what they lived and felt through meditation, clarify their doubts and solve difficulties arisen when doing home practice. |
|  |  | Remind compliance | Explain the importance of daily practice and the attendance to sessions. |
| Theory | 30 min | Group reflection on text | Through dialogue and the experiences of each person, an exchange and construction of new knowledge was generated. |
|  |  |  | Used text: Instructions and attitudes for practice^a .^ Stress in everyday life^b.^ Stress at work^b.^ Scientific evidence^c^. Applications in mental health clinical practice^d^. |
| Practice | From 15-20 to 40-45 min | Body or mind-centred practices | Practice was increasing with the weeks. Consult Table S2 for time and specific exercise. |
| Group reflection | 15-30 min | Personal experience with the practice done | In a descriptive way (non-analytical), speaking from oneself, in the first person. |
| *NOTES:*  *^a^* Simón, V. (2011). *Aprender a practicar mindfulness [Learn to practice mindfulness].* Sello Editorial  ^b^ Kabat-Zinn, J. (2009). *Vivir con plenitud las crisis [Full catastrophe living]* (3rd ed.). Kairós  ^c^ Liétor Villajos, N., Fortis Ballesteros, M., & Moraleda Barba, S. (2013). Mindfulness en medicina [Mindfulness in medicine]. *SAMFyC*, 14(2), 166-179.  ^d^ Hervás, G., Cebolla, A., & Soler, J. (2016). Intervenciones psicológicas basadas en mindfulness y sus beneficios: estado actual de la cuestión [Mindfulness-based psychological interventions and their benefits: current state of affairs]. *Clinica y Salud*, 27(3), 115-124. | | | |

| **Table S2**. Practices per session. Body-centred intervention | | | |
| --- | --- | --- | --- |
| Nº of session | Practice 1 | Practice 2 | Home practice |
| 1 | Raisin exercise (5 min) *(it is only carried out if it is the first intervention)* | Brief body scan (15 min) | Formal practice: body scan  Informal practice: mindful eating *(it is substituted by other informal practice*  *if it is the second intervention)* |
| 2 | Body scan (20 min) | Mindful stretching lying down (20 min) | Formal practice: body scan/ mindful stretching lying down  Informal practice |
| 3 | Body scan (20 min) | Mindful stretching standing (25 min) | Formal practice: body scan/ mindful stretching standing  Informal practice |
| 4 | Body scan (20 min) | Mindful stretching lying down and standing (30 min) | Formal practice: body scan/ mindful stretching lying down and standing  Informal practice |
| 5 | Body scan (30 min) | Mindful stretching (30 min) without guide, as the person chooses | Free formal practice of those practiced in the intervention  Informal practice |
| *NOTES:*  Body scan: consists of directing the attention to each body part, one at a time, from toe to head, feeling each part, normally performed lying down.  Mindful stretching: based on Hatha yoga, has a variety of postures to adopt. It is about doing stretching and smooth and slow movements, really paying attention to every change and pose you do.  Informal practice: mindful eating, mindful walking or practicing mindfulness of other routine daily activities (e.g. bathing, cleaning, etc.). | | | |

| **Table S3.** Practices per session. Mind-centred intervention | | | |
| --- | --- | --- | --- |
| Nº session | Practice 1 | Practice 2 | Home practice |
| 1 | Raisin exercise (5 min)  *(it is only carried out if it is the first intervention)* | Mindfulness of breath meditation (15 min) | Formal practice: mindfulness of breath meditation  Informal practice: Mindful eating *(it is substituted by other informal practice*  *if it is the second intervention)* |
| 2 | Mindfulness of breath meditation (20 min) | Mindfulness of sounds meditation (20 min) | Formal practice: breathing/sounds  Informal practice |
| 3 | Mindfulness of thoughts and feelings meditation (20 min) | Mindfulness of breath and sounds meditation (25 min) | Formal practice: breathing and sounds  Informal practice |
| 4 | Completed meditation (30 min) | Open awareness meditation (20 min) | Formal practice: completed/open awareness  Informal practice |
| 5 | Completed meditation 40 min) | Open awareness meditation (20 min) | Free formal practice of those practiced in the intervention  Informal practice |
| *NOTES*:  Mindfulness of breath meditation: the focus of attention is on the feeling of your in-breath and out-breath.  Mindfulness of sounds meditation: the focus of attention is the external or internal sounds that arise.  Mindfulness of thought: awareness of thoughts arising in the mind.  Mindfulness of feeling: awareness of emotions arising in the mind.  Complete meditation: mixed the previous meditation in this order: breathing, sounds, thoughts and feelings.  Open awareness meditation: awareness of sensations, thoughts or feelings without an order, as they arise in consciousness.  Informal meditation practices: mindful eating, mindful walking or practicing mindfulness of other routine daily activities (e.g. bathing, cleaning, etc.). | | | |
